# A Synthetic SARS-CoV-2-Derived T-Cell and B-Cell Peptide Cocktail Elicits Full Protection against Lethal Omicron BA.1 Infection in H11-K18-hACE2 Mice

**DOI:** 10.1128/spectrum.04194-22

**Published:** 2023-03-13

**Authors:** Yang Song, Hongqiao Hu, Kang Xiao, Xinghu Huang, Hong Guo, Yuqing Shi, Jiannan Zhao, Shuangli Zhu, Tianjiao Ji, Baicheng Xia, Jie Jiang, Lei Cao, Yong Zhang, Yan Zhang, Wenbo Xu

**Affiliations:** a NHC Key Laboratory of Medical Virology and Viral Diseases, National Institute for Viral Disease Control and Prevention, Chinese Center for Disease Control and Prevention, Beijing, China; National Institutes of Health

**Keywords:** COVID-19, Omicron variant, SARS-CoV-2, T-cell epitope, peptide vaccine, synthetic peptides

## Abstract

Emerging variants of severe acute respiratory syndrome coronavirus 2 (SARS-CoV-2) have been developing the capacity for immune evasion and resistance to existing vaccines and drugs. To address this, development of vaccines against coronavirus disease 2019 (COVID-19) has focused on universality, strong T cell immunity, and rapid production. Synthetic peptide vaccines, which are inexpensive and quick to produce, show low toxicity, and can be selected from the conserved SARS-CoV-2 proteome, are promising candidates. In this study, we evaluated the effectiveness of a synthetic peptide cocktail containing three murine CD4^+^ T-cell epitopes from the SARS-CoV-2 nonspike proteome and one B-cell epitope from the Omicron BA.1 receptor-binding domain (RBD), along with aluminum phosphate (Al) adjuvant and 5′ cytosine-phosphate-guanine 3′ oligodeoxynucleotide (CpG-ODN) adjuvant in mice. The peptide cocktail induced good Th1-biased T-cell responses and effective neutralizing-antibody titers against the Omicron BA.1 variant. Additionally, H11-K18-hACE2 transgenic mice were fully protected against lethal challenge with the BA.1 strain, with a 100% survival rate and reduced pulmonary viral load and pathological lesions. Subcutaneous administration was found to be the superior route for synthetic peptide vaccine delivery. Our findings demonstrate the effectiveness of the peptide cocktail in mice, suggesting the feasibility of synthetic peptide vaccines for humans.

**IMPORTANCE** Current vaccines based on production of neutralizing antibodies fail to prevent the infection and transmission of SARS-CoV-2 Omicron and its subvariants. Understanding the critical factors and avoiding the disadvantages of vaccine strategies are essential for developing a safe and effective COVID-19 vaccine, which would include a more effective and durable cellular response, minimal effects of viral mutations, rapid production against emerging variants, and good safety. Peptide-based vaccines are an excellent alternative because they are inexpensive, quick to produce, and very safe. In addition, human leukocyte antigen T-cell epitopes could be targeted at robust T-cell immunity and selected in the conserved region of the SARS-CoV-2 variants. Our study showed that a synthetic SARS-CoV-2-derived peptide cocktail induced full protection against lethal infection with Omicron BA.1 in H11-K18-hACE2 mice for the first time. This could have implications for the development of effective COVID-19 peptide vaccines for humans.

## INTRODUCTION

Severe acute respiratory syndrome coronavirus 2 (SARS-CoV-2) Omicron variants and subvariants with enhanced transmission and immune escape ability have been dominating the coronavirus disease 2019 (COVID-19) pandemic (1–4). However, as the virus continues to mutate, it is difficult to predict the emergence of novel variants with even greater immune escape ability and pathogenicity. Developed through various research and development routes, the COVID-19 vaccines currently in use, such as inactivated vaccines, viral vector vaccines, mRNA vaccines, and protein-based vaccines, are all primarily aimed at inducing humoral immunity and effective neutralizing antibodies to protect against SARS-CoV-2 infection (5–8). The global immunization efforts against COVID-19 have been successful in reducing the number of severe cases and deaths, but there are still challenges (5, 6, 9–11).

Current vaccines fail to prevent infection with and transmission of SARS-CoV-2 Omicron and its subvariants. Sera from vaccinated individuals show significantly reduced or lost neutralizing capacity of Omicron and its subvariants because of certain mutations in the spike protein that lead to a decline in neutralizing antibody-mediated protection (1, 11–16). Although vaccination is highly effective in preventing severe disease, higher-risk individuals, such as the elderly, those with underlying health conditions, and immunocompromised patients, may still be vulnerable to Omicron subvariants (17–20).

T cells play an important role in preventing SARS-CoV-2 infection and reducing COVID-19 severity. Studies have shown that cellular immune responses acquired through vaccination or previous infection are broadly maintained against different variants, including Omicron (21–27). The T-cell epitopes retained a high degree of preservation from wild type to Omicron (24, 25), and T-cell immunity can be maintained even in the absence of humoral immune responses (28–30). This explains why vaccines or previous infection provides robust protection against severe disease with Omicron, even with low levels of neutralizing antibodies (21, 22, 24, 30). The importance of SARS-CoV-2 T-cell immunity is increasingly recognized, and the induction of T-cell immunity is a central goal for COVID-19 vaccine development. Eliciting T-cell responses with peptide vaccination is more straightforward, since T lymphocytes usually recognize small linear epitope regions of 8 to 15 amino acids of these antigens presented by antigen-presenting cells (APCs) with the major histocompatibility complex (MHC) molecules, which can be mimicked by peptides (31, 32). CoVac-1, a T-cell epitopic peptide-based vaccine candidate developed by University Hospital Tübingen, which consists of SARS-CoV-2 human leukocyte antigen DR (HLA-DR) T-cell epitopes from different viral proteins, is currently undergoing phase II clinical trials and considered the most promising COVID-19 T cell vaccine (18).

In addition to T-cell peptides, the composition of peptide vaccines can also contain B-cell peptides to provide humoral immune responses. Synthetic linear B-cell epitopes usually have weak immunogenicity and require modification to improve their efficacy (33). However, in rare cases, some linear epitopes can fold into a three-dimensional structure and effectively induce neutralizing antibodies (33, 34); such B-cell epitopes are what we aim for. Another big advantage is that unique epitopes can be selected to avoid autoimmune responses that could be generated by the whole protein (33, 35).

To develop a safe and effective COVID-19 vaccine, it is important to understand critical factors and avoid disadvantages. This includes (i) an effective and durable cellular response, (ii) minimal effects of viral mutations, (iii) rapid production against emerging variants, and (iv) good safety and low toxicity. Synthetic peptide-based vaccines offer an excellent alternative to meet these criteria. First, they can target SARS-CoV-2-derived HLA T-cell epitopes to promote robust T-cell immunity. Second, functional T-cell epitopes can be selected from the conserved regions of the SARS-CoV-2 variant proteome, and B-cell epitopes can be modified and updated over time according to the current circulating variants. Third, peptides are inexpensive and quick to produce and have robust design procedures. Finally, peptides are very safe, exhibiting very low toxicity and lacking infectious and secondary adverse reactions (33–36).

However, the immunogenicity of peptide vaccines is relatively poor and requires good adjuvants. 5′ cytosine-phosphate-guanine 3′ oligodeoxynucleotide (CpG-ODN; referred to here as CpG) is a Toll-like receptor 9 (TLR9) agonist that can enhance the maturation of dendritic cells (DCs) to induce a Th1-cellular response. CpG has been applied to herpes simplex virus and numerous cancer vaccines, and while it can easily diffuse away from the injection site, it is well adsorbed to aluminum (Al) adjuvants (37–39).

In this study, we selected four epitopes from the Immune Epitope Database (IEDB [http://www.iedb.org/]), including three murine CD4^+^ T-cell epitopes for C56BL/6J mice and one B-cell epitope that has been reported to elicit neutralizing antibodies against wild-type SARS-CoV-2 (40). The T-cell epitopes were conserved among SARS-CoV-2 variants and were derived from nonspike proteins (41). The amino acid sequence of the B-cell epitope was modified according to the Omicron BA.1 variant. To date, no studies have reported that vaccination with a synthetic SARS-CoV-2-derived T- and B-cell peptide cocktail could confer full protection in lethally SARS-CoV-2-infected K18-hACE2 mice (42). Here, we evaluated the immunogenicity and protection against the Omicron BA.1 variant induced by administering a synthetic SARS-CoV-2-derived peptide vaccine cocktail combined with CpG and Al adjuvants in murine models.

## RESULTS

### Selection strategy of the Omicron B-cell epitope and murine T-cell epitopes.

We used the public resource from the IEDB (as of April 2022) for peptide selection. For B-cell epitope selection, the “neutralization” assay from “Biological activity” in the “Assay” section was restricted. The retrieved results of peptide sequences were from 14 publications, and we studied the experimental data from these publications for further selection. Aparicio et al. reported that the peptide from position 446 to 480 and its cyclic version from 446 to 488 (with a disulfide bridge between cysteines 480 and 488) from the receptor-binding domain (RBD) of the S protein could induce polyepitopic antibody responses in mice and display neutralizing capacity (40). The cyclic version (446 to 488) was adopted in this study and modified with 4 amino acid mutations according to Omicron BA.1 (G446S, S477N, T478K and E484A; BA1-S-446-488cc-B). For CD4^+^ T-cell epitope retrieval, “Mouse” from the “Host” section, “T cell” from the “Assay” section and “Class II” from the “MHC restriction” section were restricted. After studying the related 7 publications, three I-A^b^–restricted CD4^+^ T-cell epitopes (C57BL/6 mice) identified by Zhuang et al. from nonspike proteins were selected (N-9-23-T, ORF3a-266-280-T, and ORF7a-62-76-T) (41).

### B-cell epitope induced peptide and RBD-binding and neutralizing antibodies against Omicron BA.1.

**FIG 1:**
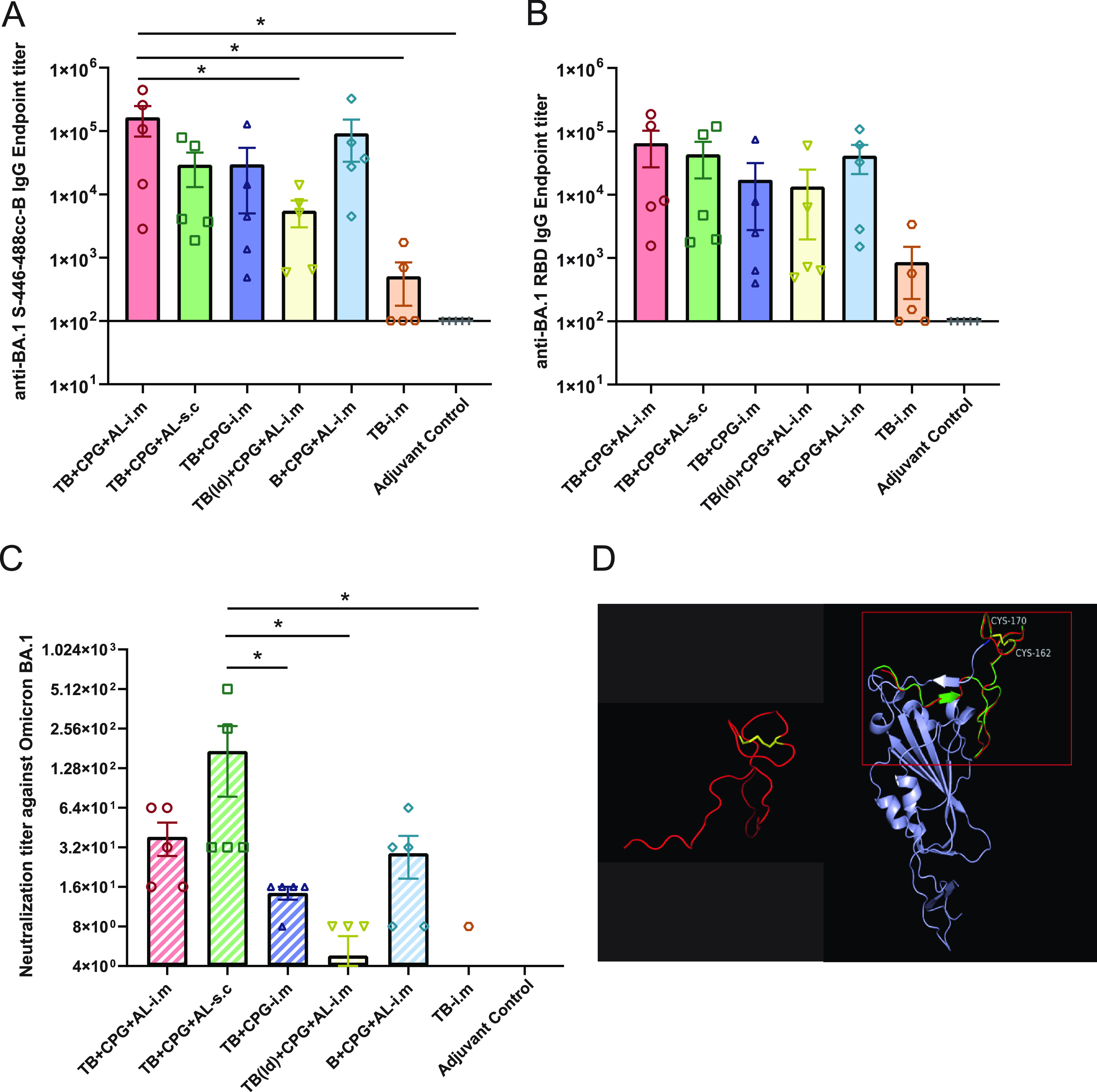
B-cell epitope-induced peptide and RBD-binding and neutralizing antibodies against Omicron BA.1. C57BL/6J mice immunized i.m. or s.c. with BA1-S-446-488cc-B with or without CpG^+^Alum/CpG adjuvants exhibited humoral responses that recognized BA1-S-446-488cc-B (A), BA.1 RBD (B), and neutralized BA.1 live virus (C). Sera were obtained at day 35 (14 days post-booster immunization) and tested against peptides BA1-S-446-488cc-B and BA.1 RBD by ELISA and BA.1 virus with a neutralization test. Points represent individual mice. For binding antibody, bars represent geometric means with endpoint antibody titers. For neutralizing antibody, the limitation of detection was the initial dilution (1:4). Statistically significant differences were measured by the appropriate one-way ANOVA. *, *P* < 0.05. The results are means and standard errors of the means (SEM) for individual mice. (D) Prediction of the conformation of the BA1-S-446-488cc-B epitope and BA.1 RBD using AlphaFold.

Serum samples were collected on day 14 after the second immunization for binding and neutralizing antibody tests. Table 1 shows animal groups and immunizations. The B-cell epitope induced the highest levels of self-binding and BA.1 RBD-binding IgG in the TB^+^CpG^+^Al-i.m. group, with average titers of 1.7 × 10^5^ and 6.5 × 10^4^, respectively. All immune groups, except the TB-i.m. group (antigen control), had some level of self-binding and RBD-binding IgG titers (Fig. 1A and B). However, when titrated with BA.1 virus, the highest neutralizing antibody titers were induced in TB^+^CpG^+^Al-s.c. group, with a geometric mean titer (GMT) of 84.5. The inconsistency between the binding IgG and neutralizing antibody titers reflected a better immune response from subcutaneous immunization in mice (Fig. 1C). This is likely due to a stronger Th response generated by CD4^+^ T-cell epitopes with subcutaneous injection, which promotes the production of effective antibodies. Furthermore, we predicted the native conformation of the BA1-S-446-488cc-B epitope using AlphaFold (Fig. 1D). The epitope exhibited a natural conformation that matched this region of the RBD almost exactly, which explains the generation of effective neutralizing antibodies.

**TABLE 1:** Animal groups and immunizations

Group	No.	Dose (μg) of:				Immunization route
		B-cell epitope	T-cell epitopes	CpG	Al	
TB+CpG+Al-i.m.	5	100	30 (each)	40	100	i.m.
TB+CpG+Al-s.c.	5	100	30 (each)	40	100	s.c.
TB+CpG-i.m.	5	100	30 (each)	40		i.m.
TB(ld)+CpG+Al-i.m.^*a*^	5	50	30 (each)	40	100	i.m.
B+CpG+Al-i.m.	5	100		40	100	i.m.
TB-i.m.	5	100	30 (each)			i.m.
Adjuvant control	5			40	100	i.m.

a ld, low dose.

### Subcutaneous immunization in mice induced the strongest Th1-biased T-cell responses.

**FIG 2:**
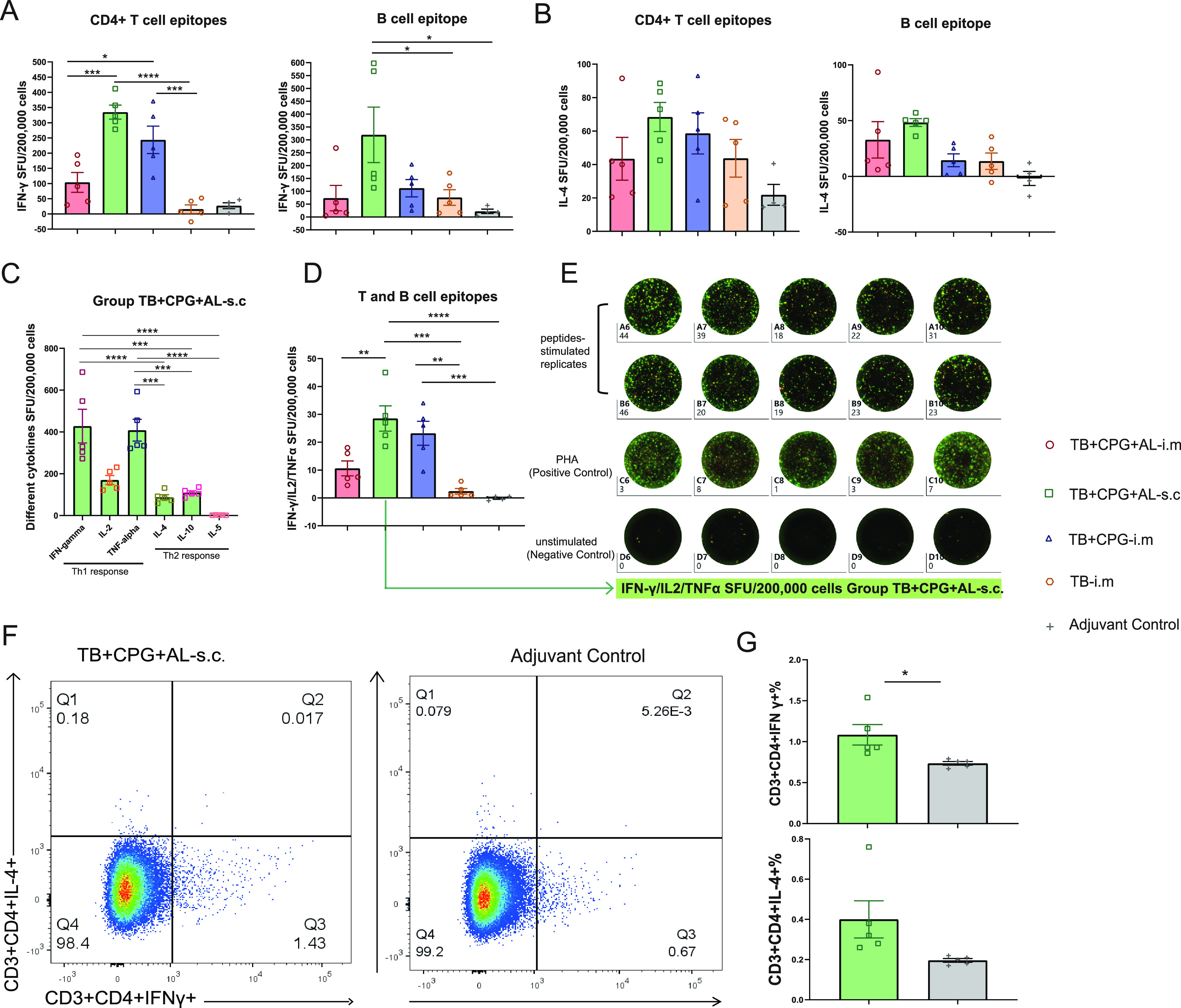
C57BL/6J mice exhibited good Th1-biased T-cell responses. C57BL/6J mouse splenocytes harvested 14 days post-booster immunization were stimulated with CD4^+^ T-cell epitopes and B-cell epitopes for (A) IFN-γ spot-forming unit (SFU) and (B) IL-4 SFU detection, respectively. (C) SFU numbers of IFN-γ, IL-2, TNF-α, IL-4, IL-10, and IL-5 in mice from the TB^+^CpG^+^Al-s.c. group stimulated with the T- and B-cell epitope mixture. (D) Numbers of cells that secreted all three Th1-biased analytes (IFN-γ, IL-2, and TNF-α). (E) FluoroSpot wells. (F) Flow cytometry analysis and (G) comparison of CD4^+^ T-cell peptide-specific CD3^+^ CD4^+^ IFN-γ^+^ T cells and IL-4^+^ cells of the TB^+^CpG^+^Al-s.c. and control groups. *, *P* < 0.05; **, *P* < 0.01; ***, *P* < 0.001; ****, *P* < 0.0001.

Mouse splenocytes harvested 14 days post-booster immunization were stimulated with CD4^+^ T-cell epitopes (gamma interferon [IFN-γ] and interleukin 4 [IL-4]), B-cell epitopes (IFN-γ and IL-4), or T- and B-cell epitope mixtures (IFN-γ, IL-2, tumor necrosis factor alpha [TNF-α], IL-4, IL-10, and IL-5) for the detection of Th1 (IFN-γ, IL-2, TNF-α)- and Th2 (IL-4, IL-10, IL-5)-biased responses of peptide-specific cell-mediated immunity (Fig. 2). In the TB^+^CpG^+^Al-s.c. group, significantly more IFN-γ-secreting cells were induced, stimulated by both the CD4^+^ T-cell epitopes and the T- and B-cell epitopes, than in the TB^+^CpG^+^Al-i.m. group (Fig. 2A; also, see Fig. S1a in the supplemental material), indicating stronger T-cell immunity with subcutaneous injection. In addition, the IFN-γ- and IL-4-secreting cells with B-cell epitope stimulation suggested that the B-cell epitope contained murine T-cell epitopes (Fig. 2A and B). Furthermore, in the TB^+^CpG^+^Al-s.c. group, the numbers of T/B epitope-specific IFN-γ- and TNF-α-secreting cells were significantly higher than the numbers of IL-4-, IL-10-, and IL-5-secreting cells, with almost undetectable IL-5-secreting cells, indicating a multifunctional Th1-biased and balanced Th2 cell-mediated immune response (Fig. 2C). In the TB^+^CpG^+^Al-s.c. group, the number of cells that secreted all three Th1-biased analytes (IFN-γ, IL-2, and TNF-α) was significantly higher (Fig. 2D), as seen in the FluoroSpot wells (Fig. 2E). Flow cytometry detected significantly more CD4^+^ T-cell epitope-specific IFN-γ in the TB^+^CpG^+^Al-s.c. group than in the control group, suggesting the presence of peptide-specific IFN-γ^+^ CD4^+^ T cells (Fig. 2F and G). The consistency between the FluoroSpot and neutralization results in the TB^+^CpG^+^Al-s.c. group indicated that a stronger CD4^+^ T-cell response could enhance the effective humoral immune response.

### Subcutaneous vaccination with peptides induces full protection in H11-K18-hACE2 mice lethally infected with Omicron BA.1.

**FIG 3:**
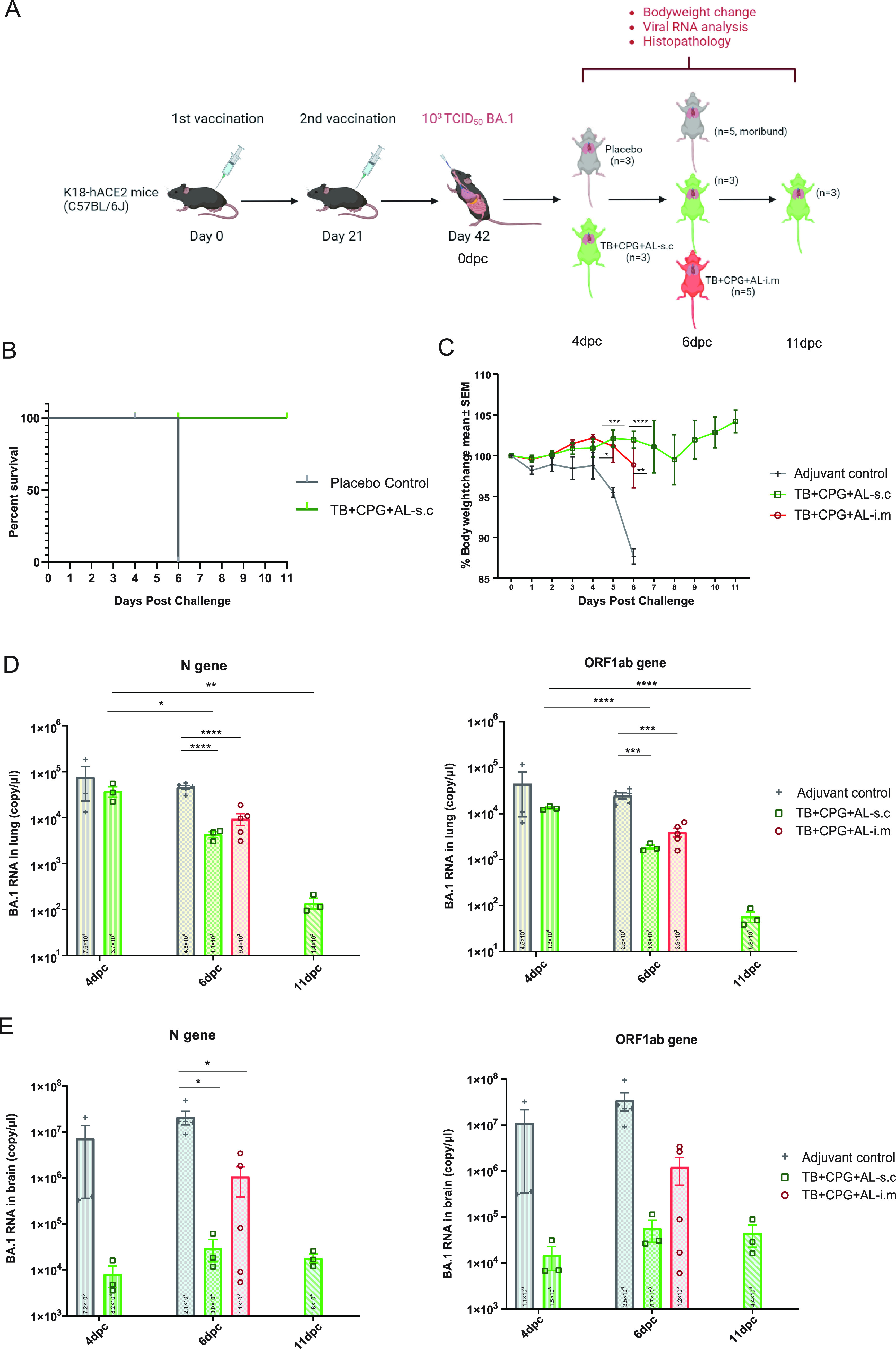
Vaccination with a mixture of B-cell and CD4^+^ T-cell epitopes protects against Omicron BA.1 challenge. (A) Schematic representation of the challenge schedule. H11-K18-hACE-2 mice were subcutaneously (*n* = 10) or intramuscularly (*n* = 5) vaccinated with a mixture of T and B peptides or adjuvant buffer (*n* = 8) on days 0 and 21 and challenged with 1,000 TCID_50_/mouse of the Omicron BA.1 strain on day 42 (one of the mice in the TB^+^CpG^+^Al-s.c. group was dead at 1 dpc, probably due to mechanical asphyxia). Following challenge, 3 to 5 mice were sacrificed for pulmonary histological and viral load analyses at 4, 6 and 11 dpc, except for the 5 mice in the TB^+^CpG^+^Al-i.m. group, which were sacrificed together at 6 dpc. (B) Mouse survival rate. (C) Change in body weight after challenge. (D and E) Copy numbers of Omicron BA.1 viral RNA of N and ORF1ab genes in lung (D) and brain (E) at 4, 6, and 11 dpc. *, *P* < 0.05; **, *P* < 0.01; ***, *P* < 0.001; ****, *P* < 0.0001.

H11-K18-hACE-2 mice were vaccinated subcutaneously (*n* = 10) or intramuscularly (*n* = 5) with T- and B-peptide cocktail compared with adjuvant control (*n* = 8) mice to evaluate the induction of protective immunity against challenge with the Omicron BA.1 strain. Mice were challenged intranasally with 1,000 median tissue culture infectious doses (TCID_50_)/mouse of BA.1 21 days after vaccination (one of the mice in the TB^+^CpG^+^Al-s.c. group died at 1 day postchallenge [dpc], probably due to mechanical asphyxia). At 4, 6, and 11 dpc, 3 to 5 mice were sacrificed for pulmonary histological and viral load analyses, except for the 5 mice in the TB^+^CpG^+^Al-i.m. group, which were sacrificed together at 6 dpc (Fig. 3A). Mice in the control group exhibited mild trembling and reduced mobility and balance at 4 dpc, which worsened on the following day. By 6 dpc, all the mice were dying or dead with approximately 12% weight loss (Fig. 3B and C). In contrast, all mice in TB^+^CpG^+^Al-s.c. group showed increased body weight with a small drop at approximately 8 dpc and survived until 11 dpc (Fig. 3B and C). The weight loss of mice in the TB^+^CpG^+^Al-i.m. group was higher than that in the TB^+^CpG^+^Al-s.c. group at 6 dpc (Fig. 3C).

In terms of virus load, at 4 and 6 dpc, mice in control group had a high level of 10^4^ to 10^5^ copies/μL in the lungs (Fig. 3D) and 10^6^ to 10^8^ copies/μL in the brain (Fig. 3E), with no significant change. At 4, 6, and 11 dpc, the TB^+^CpG^+^Al-s.c. group mice had significant decreases in viral RNA in the lungs of 10^4^, 10^3^, and 10^2^ copies/μL, respectively (Fig. 3D), and a persistent low level of 10^4^ copies/μL in the brain (Fig. 3E). When compared among the three groups at 6 dpc, the viral loads in the lungs of mice in the control group were significantly higher than those in the TB^+^CpG^+^Al-s.c. and TB^+^CpG^+^Al-i.m. groups (Fig. 3D). The TB^+^CpG^+^Al-i.m. group exhibited a higher level of viral copies in the lung and brain tissues than the TB^+^CpG^+^Al-s.c. group, which corresponded to the weight loss and previous humoral and cellular immunological results (Fig. 1C, 2, and 3D). The results of challenge of mice in the control group in our study were in accordance with the study by Lu et al., which used the same K18-ACE2 murine model with the same challenge dose of Omicron virus (43).

**FIG 4:**
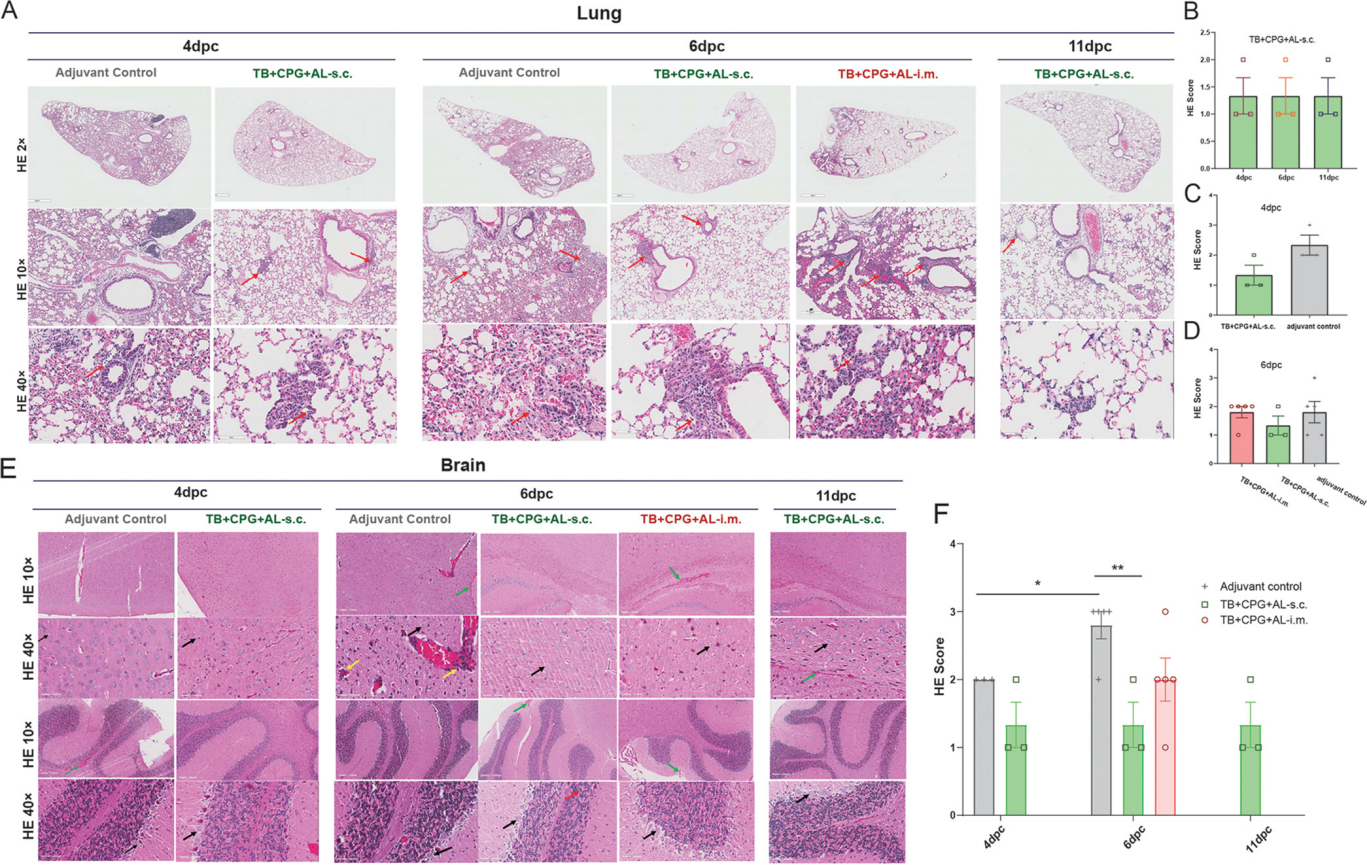
Vaccination with a mixture of B-cell and CD4^+^ T-cell epitopes reduces pathological changes in mice. (A) Lung histopathological analysis from adjuvant control- and peptide mixture-treated H11-K18-hACE2 mice at 4, 6, and 11 dpc. Arrows indicate inflammatory cell infiltrate dominated by lymphocytes. H&E staining. Bars: 2×, 800 μm to 1,000 μm; 10×, 200 μm; 40×, 60 μm. (B to D) H&E staining scores of the s.c. group at 4, 6, and 11 dpc, (B) the s.c. and adjuvant control groups at 11 dpc (C), and s.c., i.m., and adjuvant control groups at 6 dpc (D). Sections were scored on a severity scale of 0 4 according to the degree of inflammatory cell infiltration in different anatomic site of lung (0, infiltration of 0 or 1 inflammatory cell was visible; 1, infiltration of fewer than 5 inflammatory cells was visible, 2, infiltration of 5 to 10 inflammatory cells was visible; 3, infiltration of 10 to 20 inflammatory cells was visible; 4, infiltration of 20 inflammatory cells was visible). The lung disease at different anatomic sites examined by light microscopy was assessed by the expansion of the parenchymal wall, interstitial pneumonia, alveolitis, and bronchiolitis. (E) Histopathological analysis of H&E-stained brain from adjuvant control and peptide mixture-treated H11-K18-hACE2 mice at 4, 6, and 11 dpc. Black arrows indicate swelling and necrosis of neurons in the brain, cerebellum, and part of the brain stem; yellow arrows indicate localized vascular congestion and infiltration of surrounding inflammatory cells and glial cells, forming a “vascular sleeve”; green arrows indicate cortical, ventricular, and submeningeal congestion and hemorrhage. Bars: 10×, 200 μm; 40×, 60 μm. (F) H&E staining scores of all the groups at 4, 6, and 11 dpc. Sections were scored on a severity scale of 0 to 4 according to the degree of inflammatory cell infiltration in different anatomic sites of the brain (0, no abnormality was seen; 1, lesion was not obvious [between normal and abnormal]; 2, lesions were observable but not yet severe; 3, significant lesions with potential for more severe development were seen; 4, lesions over large areas of tissue and organs were seen). *, *P* < 0.05; **, *P* < 0.01.

Histological analysis was performed at 4, 6, and 11 dpc. At 4 dpc, mice in control group presented a thickening of the alveolar septa and interstitial inflammation with hemangiectasis, hyperemia, interstitial edema, and lymphocyte and monocyte infiltration (Fig. 4A). In contrast, mice in the TB^+^CpG^+^Al-s.c. group displayed a reduction in pathological reactions and lung inflammation at 4 and 6 dpc, suggesting that TB^+^CpG^+^Al-s.c. may delay disease progression with milder lung inflammation during Omicron BA.1 infection (Fig. 4B to D). Moreover, brain tissues from the control group presented severe lesions, manifested as neuronal swelling, necrosis, and focal hemorrhage, and some samples showed a “vascular mantle” and hippocampal nerve cell swelling (Fig. 4E and F). However, the TB^+^CpG^+^Al-s.c. group showed significantly milder lesions. In addition, the TB^+^CpG^+^Al-i.m. group displayed more severe lesions in both lung and brain tissues than the TB^+^CpG^+^Al-s.c. group at 6 dpc. The above results were in line with the viral copy results showing that mouse immunization induced neutralizing antibodies and CD4^+^ cellular response against Omicron BA.1 and protected H11-K18-hACE-2 mice against infection, with a reduction in viral load and pathological changes, and subcutaneous injections may provide better protection.

## DISCUSSION

In this study, we investigated the effectiveness of a two-dose subcutaneous vaccination consisting of synthetic single B cells and three CD4^+^ T-cell epitopes derived from SARS-CoV-2. We found that this peptide cocktail vaccination provided full protection against lethal Omicron BA.1 infection in H11-K18-hACE2 mice, with a reduction in viral load and pathological changes. Notably, this is the first study to demonstrate that a mixture of simple synthetic peptides can protect against a lethal dose of SARS-CoV-2 in a murine model, with just one booster dose. A previous study (available as a preprint [44]) used a peptide-based vaccine cocktail, consisting of 16 synthesized SARS-CoV-2-derived T and B peptides combined with TLR-based adjuvants with one booster dose. However, that study failed to provide any protection against infection with the murine-adapted virus SARS-CoV-2-MA10 or against disease following infection (44). Apart from no production of neutralizing antibodies, the authors of that study discuss three possible scenarios for this failure, including the ideas that T-cell responses do not work for murine adapted virus, that there is a significant mismatch between T-cell epitopes and BALB/c mice, and that T-cell responses do not play a protective role against SARS-CoV-2 in the absence of antibody responses. However, the authors themselves refuted the last possibility, given the contradictory findings of many previous animal studies. In our study, we not only observed effective neutralizing antibodies but also refuted the first two scenarios.

Our results may again emphasize the essential role of cellular immunity, particularly CD4^+^ T-cell immunity, in preventing SARS-CoV-2 infection. While a B-cell epitope can produce some level of neutralizing antibodies against the Omicron BA.1 variant, its relatively weak immunogenicity means that it is not as effective as other types of COVID-19 vaccines. Only a high dose of 100 μg B-cell epitope per mouse was able to produce neutralizing antibodies, and reducing the dose to 50 μg resulted in the failure to produce such antibodies. This suggests that the three CD4^+^ T-cell epitopes may play an influential role in viral challenge, as CD4 T helper cells not only help with the specific antibody induction of the B-cell epitope but also induce more permanent immune control and epitope spreading. CD4^+^ T cells play a crucial role in effective cellular immunity, and studies have indicated that the selection of HLA class II-restricted antigen targets recognized by CD4^+^ T cells is of paramount importance for clinical outcome (45–49). CD4^+^ T cells support B-cell responses in the germinal center, and some CD4^+^ T cells also have direct antiviral properties similar to those of CD8^+^ T cells (48, 50–52). Furthermore, the FluoroSpot results indicated that the B-cell epitope may contain murine CD8^+^ T-cell epitopes, and the CD8^+^ T cells were expanded and survived due to the function of effective CD4^+^ T cells.

Peptide vaccines and therapeutics are typically given via subcutaneous injection. Clinical trials of COVID-19 T-cell peptide vaccines have used both subcutaneous and intradermal injection routes (18, 53). Studies show that better T-cell responses are generated after two subcutaneous injections than intramuscular injections of the BNT162b2 vaccine (54). In research on mice, subcutaneous immunization at the base of the tail induced stronger T-cell immunity. This injection route is called intradermal (i.d.) in some studies as well, because there is very limited subcutaneous space under the skin of the tail.

Subcutaneous immunization at the base of the mouse tail allows the vaccine to be delivered directly to skin dendritic cells, resulting in better cellular immunity, which further exemplified the role of the CD4^+^ T-cell response (55–57). More antigen-presenting cells, especially DCs, can more effectively present MHC-polypeptide complexes to CD4^+^ T cells and induce stronger antiviral immune responses and higher titers of neutralizing antibodies. Additionally, a Th1-biased immune response counters the theoretical risk of vaccine-associated enhanced respiratory disease, which has been associated with a Th2-driven immune response.

T-cell vaccines for COVID-19 are effective in preventing infection, and specific peptide combinations can activate SARS-CoV-2-reactive T cells (18, 21–23). Furthermore, conserved proteins can be used to select effective T-cell epitopes for a universal and antimutation vaccine strategy. The published data of CoVac-1 from phase I trial indicated that the CoVac-1 vaccine candidate has a favorable safety profile and induces potent T-cell responses after a single subcutaneous vaccination (18), and a phase I/II trial reported promising T-cell responses and safety in patients with cancer or immunoglobulin deficiency (32). CoVepiT and Pep-GNP-SARS-CoV-2 are both HLA-I T-cell multiepitopic peptide vaccines in phase I clinical trials (53). The above vaccine candidates demonstrated the feasibility of T-cell-based peptide vaccines, which is a large step forward in the field of vaccines for infectious diseases, as the approach of T-cell epitopic peptide vaccines has been mostly used in cancer therapy (58, 59).

To create an effective T-cell peptide vaccine for SARS-CoV-2, it is important to select SARS-CoV-2-derived HLA allotypes that cover a larger portion of the population. Common HLA allotypes can be selected using computational techniques and bioinformatics resources, such as the SYFPEITHI and NetMHCpan (60–63) algorithms, and mutation-prone regions should be removed. Many functional SARS-CoV-2-derived T-cell epitopes have been identified and require further experimental validation (64–66). For effective B-cell epitope selection, the three-dimensional structure of the epitope is crucial. Short peptide sequences risk losing or distorting the three-dimensional (3D) structure, so selecting a linear epitope that targets the surface of a viral receptor and maintains its natural conformation is necessary. The BA1-S-446-488cc-B epitope has a natural conformation that matches the RBD region, making it effective at generating neutralizing antibodies. The use of suitable adjuvants, such as CpG and aluminum, can enhance and regulate the immune response (39, 67, 68).

Whereas CD4^+^ helper T cells orchestrate the immune response and enable B cells to produce antibodies, CD8^+^ cytotoxic T cells eliminate virus-infected cells. Studies have reported that peptide-based vaccines targeting MHC class I epitopes can provide protection against SARS-CoV-2 infection in nonhuman primates (69), and a third vaccination with a single CD8^+^ T-cell epitope conferred protection in a K18-ACE2 model of SARS-CoV-2 infection (70). Selecting optimal antigens for a vaccine raises questions about ideal peptide length, numbers of peptides, and CD4^+^ versus CD8^+^ T-cell epitopes. HLA allotype restriction of HLA I-presented peptides can be overcome by using promiscuous CD4^+^ T-cell epitopes with multiple HLA class I peptides. The CoVac-1 vaccine adopted this strategy with success (18); the six SARS-CoV-2 HLA-DR T-cell epitopes chosen are all embedded with HLA I-binding epitopes, and the published data indicated the induction of both CD4^+^ and CD8^+^ T-cell responses.

Increasing numbers of scholars and vaccine development strategists recognize the feasibility of T-cell-activating peptides; they have employed a strategy of incorporating T-cell peptides in vaccines that mainly produce humoral immunity, which is a promising way to enhance T-cell immunity on the basis of good humoral immunity. UB-612 is a multiepitope subunit vaccine containing the S1-RBD-sFc protein and rationally designed promiscuous peptides representing conserved HLA-I and -II epitopes. The phase II trial results revealed long-lasting B- and broad T-cell immunity (71). Many preclinical vaccine candidates have focused more on T-cell immunity (53). A deeper understanding of the role of T-cell immunity in protection against SARS-CoV-2 infection and disease should provide a foundation for improving the use of current vaccines and the development of next-generation vaccines.

This study has some limitations. Our primary idea was to evaluate a T-cell-immunity-based peptide vaccine with humoral immunity (B-cell peptide) as a support. Due to limitations in the biosafety level 3 (BSL-3) laboratory and the availability of cages, we were unable to set up experimental groups with only B-cell or T-cell peptides in K18-ACE2 mice for comparison with mixed T- and B-cell peptide groups. Further experiments will be conducted to assess the protective effects of B-cell and T-cell epitopes against Omicron variants. The primary challenge in evaluating challenge protection for HLA epitope peptides is the lack of suitable murine models. To address this, a trinal transgenic model that includes HLA-A, HLA-DR, and human ACE2 genes would be the most appropriate for testing SARS-CoV-2 challenges. We have already identified functional HLA-DR T-cell epitopes embedded with HLA-I epitopes that cover various HLA allotypes through computational methods and *in vitro* assays. We are currently working on establishing an HLA/ACE2-transgenic mouse model for future peptide vaccine evaluations.

The effectiveness of the HLA T cell peptides can be verified only in humans, because no suitable animal model exists due to the discrepancies in the MHC among species. Therefore, we used a peptide vaccine cocktail containing C57BL/6J-MHC-restricted T-cell epitopes and a B-cell epitope to observe its immunization effectiveness. The results showed that the peptide vaccine cocktail induced full protection against lethal infection with Omicron BA.1 in H11-K18-hACE2 mice. In conclusion, the conserved T-cell epitopes in the peptide vaccine remain conserved and functionally stable, and the supporting B-cell epitope peptide can be dynamically synthesized and updated according to the prevalent variant, which is the most advantageous aspect of synthetic peptide vaccine. We used nonspike T-cell peptides from the conserved region with a BA.1 B-cell peptide to validate the feasibility and rationale of their approach. The findings of this study may provide valuable data support for other scientists interested in developing COVID-19 synthetic peptide vaccines.

## MATERIALS AND METHODS

### Mice, biosafety, and ethics.

Specific-pathogen-free (SPF) C57BL/6J and K18-hACE-2 female mice (6 weeks old) were purchased from Beijing HFK Bioscience Co., Ltd., and Gem Carmatech Co., Ltd., respectively. The mice were raised in the Animal Experimental Center of the Chinese Center for Disease Control and Prevention (China CDC). All experiments on mice were approved by the Animal Ethics Committee at the China CDC. The challenge test was conducted in the animal biosafety level 3 (BSL-3) laboratory at the National Institute for Viral Disease Control and Prevention (IVDC), China CDC. All animal studies were approved by IVDC, China CDC (under ethical inspection form 220414042 and BSL-3 animal experimental ethical inspection forms 122053014 and 122053016) and were carried out in strict accordance with the recommendations in the *Guidelines for the Care and Use of Laboratory Animals* (72).

### Peptides, adjuvants, and C57BL/6J mouse vaccination.

Peptides of SARS-CoV-2 Omicron BA.1 B-cell epitope BA1-S-446-488cc-B (SGNYNYLYRLFRKSNLKPFERDISTEIYQAGNKPCNGVAGFNC, containing a disulfide bridge between cysteines 480 and 488) and conserved murine T-cell epitopes N-9-23-T (QRNAPRITFGGPSDS), ORF3a-266-280-T (EPIYDEPTTTTSVPL), and ORF7a-62-76-T (QFAFACPDGVKHVYQ) were synthesized by Beijing BioAct Peptide Biotechnology Co., Ltd., with a purity of >95%, as analyzed by high-performance liquid chromatography (HPLC). C57BL/6J mice were randomly divided into 7 groups with 5 mice in each group and administered different peptide combinations with or without CpG (ODN; Changchun Huapu Biotechnology Co., Ltd.) and aluminum phosphate (Croda) (Table 1). Mice in each group were immunized twice with different components dissolved in sterilized water of a total volume of 100 μL per dose injected subcutaneously (s.c.) in the tail base or intramuscularly (i.m.) on days 0 and 21. Mice were euthanized on day 35 for the collection of the blood and spleens, which were later used for immune response assays. Related patents for the BA1-S-446-488cc-B related peptides are currently pending under the patent number 202211581104.7.

### Antigen-binding ELISA and model prediction.

Enzyme-linked immunosorbent assays (ELISAs) were performed to determine the antibody titers induced by the B-cell epitope (BA1-S-446-488cc-B) in C57BL/6J mouse sera. Ninety-six-well ELISA plates (Thermo Fisher, Denmark, 442404) were coated with BA1-S-446-488cc-B (2 μg/mL; 100 μL/well) or Omicron RBD protein (40592-V08H121-100; SinoBiological, China) (1 μg/mL; 100 μL/well) in ELISA coating buffer (R20934; Shanghai Yuan Ye Bio-Technology Co., Ltd., China) (500 mL) at 4°C overnight. The plates were washed four times with phosphate-buffered saline containing 0.05% Tween 20 (PBST; 1,000 mL) (R40025; Shanghai Yuan Ye Bio-Technology Co., Ltd., China) and then blocked with 2% bovine serum albumin (BSA) at room temperature (RT) for 1 h. Serum collected on day 35 was continuously diluted 5 and 4 times from 1:100 for BA1-S-446-488cc-B and Omicron RBD, respectively, added to each well (100 μL/well), and incubated for 1 h at RT. The plates were again washed and then incubated with a 1:5,000 dilution of horseradish peroxidase (HRP)-conjugated goat anti-mouse immunoglobulin secondary antibody (ZB-2305; SolelyBio, China) for 1 h at RT. The reaction was terminated with 2 M hydrochloric acid after addition of 3,3′,5,5′-tetramethyl dihydrochloride (TMB; Thermo Fisher Scientific) substrate for 20 min, and the absorbance was measured at 450 nm with an enzyme plate. When the optical density at 450 nm (OD_450_) was greater than or equal to 2.1 times the serum OD_450_ of the negative mice, the value was considered positive.

The B-cell epitopic and Omicron RBD structure models were predicted using AlphaFold with the genetic database parameter used at CASP14.

### Live virus neutralization assay.

Sera from immunized C57BL/6J mice were heated at 56°C for 30 min for inactivation and then serially diluted in cell culture medium in 2-fold dilutions starting from 1:4 using 96-well plates. The diluted sera (50 μL/well) were mixed with an equal volume of solution containing 100 TCID_50_ live SARS-CoV-2 Omicron BA.1 isolate (BA.1-HK1-IVDC) in each well. After 2 h of incubation at 37°C in a 5% CO_2_ incubator, Vero-E6 cells (1 × 10^4^ cells/100 μL/well) were treated with serum and virus. Infected cells were incubated for 4 days at 37°C and 5% CO_2_, and the cytopathic effect (CPE) in each well was recorded under a microscope. The neutralization titer was calculated as the logarithm of the maximum dilution required for 50% neutralization of virus infectivity by Karber’s method.

### FluoroSpot assays and flow cytometry.

FluoroSpot assays measuring different cytokine-secreting T cells were used to determine the Th1- or Th2-biased cell responses of lymphocytes in the spleen. The mice were euthanized on day 35, and the spleen was collected aseptically and fully milled. Splenocytes were seeded at 3 × 10^5^ cells/well in three different cytokine detection FluoroSpot Plus kits, including IFN-γ and IL-4 to determine the overall T-cell response, IFN-γ, IL-2, and TNF-α to determine the Th1 response, and IFN-γ, IL-10, and IL-5 to determine the Th2 response (FSP-4146, FSP-414245, and FSP-414743; Mabtech, Sweden). Splenocytes were stimulated *in vitro* with T- and B-cell peptides (10 μg/mL for each peptide) for 36 h in an incubator at 37°C containing 5% CO_2_. The subsequent experimental procedures were performed according to the manufacturer’s instructions. The spot-forming cells (SFCs) were counted under an IRIS Mabtech ELISpot/FluoroSpot reader.

For the flow cytometry analysis, cells harvested from excised spleens were dispersed into RPMI 1640 (Gibco) through a 40-μm cell strainer using the back of a 1-mL syringe plunger. Cell mixtures were subjected to hypotonic lysis to remove red blood cells, washed twice in 2 mM (ethylenedinitrilo)tetraacetic acid and 1% fetal bovine serum (FBS) in PBS and resuspended in the same solution containing the corresponding fluorescent dye-conjugated antibodies. Staining steps were carried out at 1:100 dilutions in the presence of Fc block (BioLegend, California, USA) for 30 min at 4°C in the dark. Samples were washed twice with fluorescence-activated cell sorting (FACS) buffer before further analysis. All flow data were acquired on a FACSCanto II flow cytometer (BD Biosciences) and analyzed using FlowJo software (Tree Star). The gating strategy for flow cytometry can be found in Fig. S2.

### H11-K18-hACE-2 mouse vaccination and viral challenge.

H11-K18-hACE-2 transgenic mice (C57BL/6J background) were divided into 3 groups: the TB^+^CpG^+^Al-i.m. (experimental group 1; *n* = 5), TB^+^CpG^+^Al-s.c. (experimental group 2; *n* = 10), and CpG^+^Al-i.m. (adjuvant control group; *n* = 8) groups. The immunization procedure was the same as that for C57BL/6J mice (on days 0 and 21). Vaccinated animals were then transferred to the BSL-3 animal laboratory at IVDC on day 41 and were intranasally infected with 1 × 10^3^ TCID_50_ of Omicron BA.1 (BA.1-HK-1-IVDC, the strain is preserved in IVDC) in a total volume of 50 μL on day 42. Challenged mice were weighed every day from the day of challenge until 11 days postchallenge (dpc). Mice were sacrificed at three time points: at 4 dpc, three mice each from the TB^+^CpG^+^Al-s.c. and control groups were sacrificed; at 6 dpc, when the mice in the control group were dead and dying, five mice each from the TB^+^CpG^+^Al-i.m. and control groups and four mice from the TB^+^CpG^+^Al-s.c. groups were sacrificed; and the last 3 mice from the TB^+^CpG^+^Al-s.c. group were sacrificed at 11 dpc (assuming that the mice survived). Lungs and brains were harvested after the mice were euthanized for viral titer detection in tissue homogenate and histopathological analysis.

### SARS-CoV-2 RNA quantification in lung and brain tissues.

Lung and brain tissues were homogenized and then clarified by centrifugation at 11,000 rpm for 5 min. One hundred micrograms of supernatant was added to a nucleic acid extraction kit (Xi’an Tianlong, China). Real-time reverse transcription-PCR was performed using a nucleic acid detection kit (20210810F; Shanghai BioGerm, China), and the N and ORF1ab genes were amplified. The viral copies were quantified according to the SARS-CoV-2 nucleic acid standard substance (GBW [E] 091089; The National Institute of Metrology, China).

### Histopathological analysis.

Tissues of challenged K18-hACE-2 mice were fixed in 4% formalin and embedded in paraffin. Sections were prepared and stained with hematoxylin and eosin (H&E). Lung sections were scored on a severity scale of 0 to 4 according to the degree of inflammatory cell infiltration in different anatomic sites (0, infiltration of 0 or 1 inflammatory cell was visible; 1, infiltration of fewer than 5 inflammatory cells was visible, 2, infiltration of 5 to 10 inflammatory cells was visible; 3, infiltration of 10 to 20 inflammatory cells was visible; 4, infiltration of 20 inflammatory cells was visible). Lung disease at different anatomic sites examined under light microscopy was determined by the expansion of the parenchymal wall and the presence of interstitial pneumonia, alveolitis, and bronchiolitis. The severity of brain tissue lesions was evaluated according to their extent. Sections were scored on a severity scale of 0 to 4 according to the degree of inflammatory cell infiltration in different anatomic sites of the brain (0, no abnormality was seen; 1, lesion was not obvious [between normal and abnormal]; 2, lesions were observable but not yet severe; 3, significant lesions with potential for more severe development were seen; 4, lesions over large areas of tissue and organs were seen).

### Statistical analysis.

Statistical analyses were performed using Prism 8.0 (GraphPad Software). Comparisons were performed using ordinary one-way or two-way analysis of variance (ANOVA) with Tukey’s multiple-comparison test. *P* values of <0.05 were considered significant.
