# Supplementary material for: A Synthetic SARS-CoV-2-Derived T-Cell and B-Cell Peptide Cocktail Elicits Full Protection against Lethal Omicron BA.1 Infection in H11-K18-hACE2 Mice
Source: Microbiol Spectr. 2023 Mar 13;11(2):e04194-22. doi: 10.1128/spectrum.04194-22 (PMC10100915; doi:10.1128/spectrum.04194-22)
Supplement: Supplemental file 1 — Fig. S1 and S2. Download spectrum.04194-22-s0001.pdf, PDF file, 0.5 MB [file spectrum.04194-22-s0001.pdf]

Vaccination with a mixture of synthetic SARS-CoV-2-derived single B-cell and three CD4+ T-cell epitopes induces full protection against lethal infection with Omicron BA.1 in K18-hACE2 mice

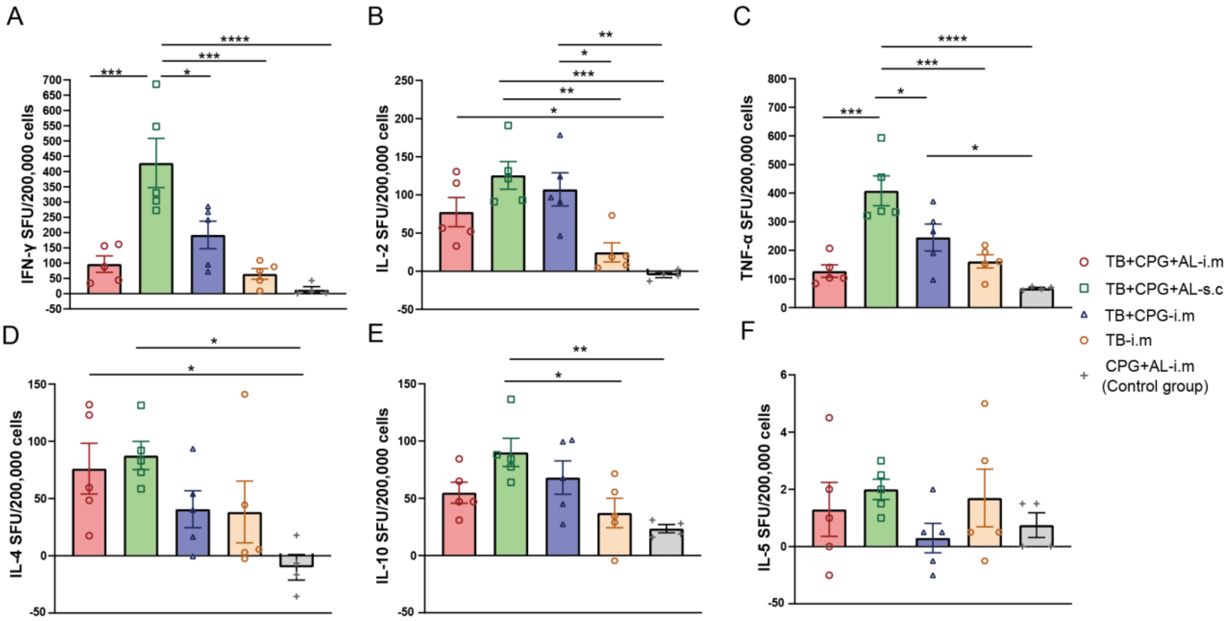

Supplementary Figure 1. C57BL/6J mouse splenocytes harvested 14 days post-booster immunization were stimulated with mixed B-cell and CD4+ T-cell epitopes for (A) IFNγ secreting forming unit (SFU), (B) IL-2 SFU, (C) TNF-α SFU, (D) IL-4 SFU, (E) IL-10 SFU and (F) IL-5 SFU detection, respectively.

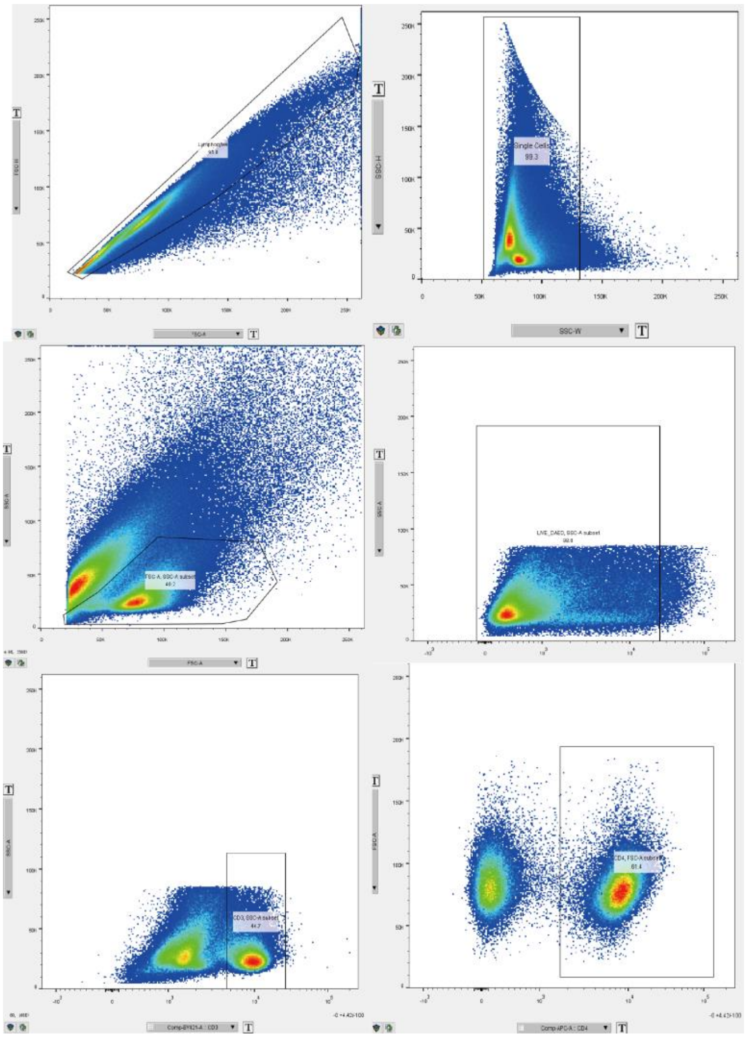

Supplementary Figure 2. Gating strategy for flow cytometry analysis. First remove adherent cells with FSC-A and FSC-H, then gate the major cell populations with FSC-A and SSC-A as axes, remove cell debris, remove dead cells with dead and live dyes, gate T cells with CD3+ and SSC, and further gate CD4 T+ cells
